# Abstract of the Proceedings of the Buffal Medical Association

**Published:** 1870-10

**Authors:** Wm. C. Phelps

**Affiliations:** Secretary


ART. II.—*Abstract of the Proceedings of the Buffal Medical Association.*

BUFFALO, October 4th, 1870.

The meeting was called to order by the President.

Dr. George D. Slocum was duly elected a member of the Association.

Dr. Potter read the following:

BUFFALO, Sept. 26, 1870.

PROF. JAMES P. WHITE:

Having now assisted you in five cases of ovariotomy, I would be much pleased, with your permission, to report the same at the next meeting of the Buffalo Medical Association.

Respectfully your obd't. serv't.,

M. G. POTTER.

BUFFALO, Sept. 26, 1870.

DR. M. G. POTTER:

I have no objection to your reporting the cases of ovariotomy in which you have assisted me, if you desire to do so. The cases referred to are in my second series of cases, the 12th, 13th, 14th, 15th and 19th. You will please so number them, and oblige

Yours very truly, JAMES P. WHITE.

CASE 12.—Acute multilocular ovarian disease; once tapped. Operation August 2, 1869, at Middleville, Herkimer County, N. Y. Recovery.

Mrs. A. T., Aet. 32; is the widowed mother of one child, now ten years of age. Until within the last six months she has been an unusually healthy and vigorous woman. In January, 1869, her abdomen began to increase in size at its lower portion, the enlargement being about central. This enlargement increased with great rapidity. On the 22nd day of May, 1869, it is said by Dr. Bushnell, of Little Falls, to be about the size of a "fœtal head, and hard." At the date of the operation, about two months later, she is much larger than a woman at full term of pregnancy. In the meantime, viz.: on the 25th day of June, 1869, she was tapped, drawing off about eight ounces of fluid. Her general health was not much impaired until about the middle of March, when she had an attack of peritonitis, lasting two weeks. Two months later she suffered from a similar attack, having a similar duration. From this time forward her health has been gradually failing. She has a strong constitution, resolute will, is determined to get rid of her burden and to survive ovariotomy.

Diagnosis, Acute Multilocular ovarian tumor; a favorable case for operative interference.

Operation August 2, 1869, at Middleville, N. Y. Physicians present: Drs W. B. Schermerhorn, attending Physician, and Varner, of Middleville; Drs. Bushnell and Sharer, of Little Falls; Millington, of Poland; Davenport, of Herkimer; Walker, of Illion and Strong, of Westfield. The anæsthetic was administered by Dr. Sharer, anæsthesia being produced by chloroform, and maintained throughout the operation by ether. An incision 4½ inches in length was made in the linea alba, through the peritoneum. An examination with the finger showed that the parietal attachments were not very firm A presenting cyst was tapped, and through this opening another larger cyst was evacuated. The fluid from the smaller cyst was rather thick and of a lightish color, while that from the larger one presented altogether a disintegrated and acrid appearance. It caused the hands of the operator to feel, for 24 hours, as if they had been immersed in a strong alkaline solution, and some of it, which was thrown into the yard, chanced to be eaten by some chickens, causing their instantaneous death. It is to be regretted that no particular examination was made of this fluid, as its absorbtion may have caused the failing health which characterized the case during the few weeks prior to the operation. The cysts having been evacuated, the adhesions were torn away, the tumor turned out, and found attached to the left ovary by a very vascular pedicle. The clamp was, however, applied and the pedicle slowly burned off with the red hot iron. Upon loosening the clamp no hemorrhage was perceptible. The abdominal cavity was then thoroughly sponged out; the pedicle returned, and the wound closed by deep sutures of silver wire and superficial sutures of silk. The usual bandage, consisting of several strips of adhesive plaster, each two inches in width and about four feet in length, was then applied and the patient carried to her bed with a pulse 98 per minute, two less than an hour before the operation. Time occupied, an half hour. Weight of tumor 35 pounds.

August 5.—Since the operation pulse has ranged from 100 to 120 per minute, regular and soft. Patient has been kept so well under the influence of opium that she has experienced no pain in the abdomen.

August 6.—Pulse 104 per minute; tongue clean; no abdominal pain; patient cheerful; kidneys acting well.

August 7–8.—Pulse 92 to 96 per minute; patient cheerful; superficial sutures removed.

August 9.—Pulse 120, soft and regular; strong inclination to evacuate the bowels, with considerable pain in the lower part of the rectum. This, upon examination, was found blocked up with impacted fæces, which copious injections succeeded in removing, much to the relief of the patient; no abdominal pain.

August 11.—Pulse 120; bowels slightly tympanitic. ℞ Olei Ricini ℥ss. Silver sutures removed; union complete.

August 12.—Pulse 98; no tympanitis; tongue clear; no abdominal pain; urine normal.

August 18.—Pulse for the last four days 80 per minute; tongue entirely clear; patient cheerful; no abdominal pain. "In fact," says Dr. Schermerhorn, from whose letters to Prof. White the history of the case after the operation, is taken, "Mrs. T. has a better looking tongue and a better pulse than she has had at any time during the last three months." Her appetite is good.

September 27, 1870.—In a letter to the writer, bearing this date, Dr. Schermerhorn says, that "Mrs. T. entirely recovered from the operation in four weeks, and has since been in almost perfect health."

Case 13.—Multilocular Ovarian Tumor; strong adhesions; tapped six times; pregnancy existing. Operation September 16, 1867, at Batavia, N. Y.; miscarriage on the tenth day. Recovery.

Mrs. D., aet. 42, was born in Germany. She is the mother of six children, youngest 8 years old. Her menstruations have always been regular and normal. She has, for the most part of her life, been healthy, vigorous and strong. Four years since, in the summer of 1865, she began to have frequent headaches, and was at the same time afflicted with severe pain in the right side. Upon examination she discovered a tumor in the right lumbar region. Soreness was at all times present in this region, and not infrequently the pain there was excruciating. For some months the patient could not determine whether the tumor increased or not. At the expiration of a year, however, it became evident to her that it was slowly becoming larger. During the next year its increase in size was still more perceptible, when she applied to a medical gentleman for relief. From this time until the spring of 1869, Mrs. D. took various prescriptions from various physicians. The tumor enlarged quite rapidly. It began to interfere with respiration. It had given rise to several attacks of peritonitis, some of which had been quite severe. Until June, menstruation had been regular and normal. In June, 1869, she was first tapped by Dr. Ganson, of Batavia, 13½ pounds of a thin, aqueous fluid escaping. From this time until September, 1869, Dr. Ganson repeated the operation four times, each time removing about the same quantity of fluid; its color and consistency, however, varying considerable. At about the first of September Mrs. D. came to Buffalo for the purpose of placing herself under the care of a German quack, residing in the suburbs of the city. While there she became very much worse and was induced to discharge the quack and apply to Dr. Tobie, who visited her on the 9th day of September and tapped her, but did not succeed in removing much of the fluid; he therefore advised variotomy. Accordingly, Prof. White was called upon.

Diagnosis: Multilocular tumor of right ovary. Operation at Batavia, September 16, 1869, at 5 P. M. Physicians present: Drs Tozier, attending Physician; L. Cotes, John Cotes, Benham, Clark and Ganson, of Batavia; Cleveland and Williams, of LeRoy; O. R. Croff and G. Croff of Bethany, and Tobie, of Buffalo.

The temperature of the room having been raised to 99° F., the patient was brought slowly under the influence of chloroform by Dr. Cleveland. Anasthesia was maintained, however, during the operation, as in all the operations for variotomy, by Prof. White, with ether. An incision four inches in length was made in the linea alba, and carried through the peritoneum. The parietal attachments were numerous but could be readily torn away. Three cysts were then in turn evacuated, which so reduced the tumor that it could be turned out and found attached to the right ovary by a long and very vascular pedicle. A small clamp was applied and the pedicle brought to the lower angle of the wound. The abdominal cavity was then gently sponged out, with as little interference as possible with the gravid womb. The wound was closed by four deep sutures of silver wire, and three superficial sutures of silk. The usual bandage was then applied, the patient carried to her bed and morphine administered subcutaneously. Time occupied, 30 minutes; weight of tumor, 37 pounds.

As consciousness returned, the patient experienced considerable pain in the abdomen, rendering the frequent repetition of the anodyne necessary. At 10 P. M. she has taken two grains of morphine since the operation, and the pain continuing the following was prescribed by Dr. Tozier, who assumes subsequent management of the case: Morphiæ Sulph. gr ss. every hour till sleep is produced. Pulse 120. ℞ brandy ℥ss every three hours.

September 17, 8 A. M.—No sleep during the night; pain diminished in severity; vomiting has occurred; pulse 114; brandy and morphia omitted. ℞ Bismuthi Sub. Nitratis gr.v every hour.

12 M.—Pulse 114; no vomiting since last night; brandy and morphia resumed.

8 P. M.—Pulse 136; has slept well.

September 18.—Pulse 126; tongue clear; takes nourishment well; brandy discontinued.

September 19.—Pulse 130, full and soft; no vomiting.

September 21.—Since last report, pulse has remained at 126 per minute; tongue clear; has passed a good night; adhesive plasters and a portion of sutures removed; union by first intention throughout the portion of the wound not occupied by the pedicle.

September 22.—Pulse 120; has slept well; retains nourishment; tongue clean.

September 23, 9 A. M.—All dressings and remaining sutures removed; union complete; plasters reapplied.

6 P. M.—Has had a chill; pulse 140; vomited three times during the day. She is given a subcutaneous injection of morphine, and ordered Quiniæ Sulp. qr. ij. every four hours.

September 24, 9 A. M.—Pulse 142; has vomited many times during the night. The retching has burst open the wound, and considerable hemorrhage has resulted; wound again closed by silk sutures.

September 26.—Has vomited most all night; pulse 150, small; has had a slight passage from the bowels; miscarried at 4 A. M. with but few pains and little flowing.

September 27.—Condition much same as at last report; considerable tympanitis. From this date the patient continued slowly to improve, complete recovery, however, not taking place till two months after the operation. In the meantime she had considerable peritoneal inflammation, attended by an irritable stomach and more or less prostration.

November 11.—Patient about the house attending to her household affairs. Recovery established.*

CASE 14.—Multilocular tumor of left ovary. Firm adhesions. Operation October 25, 1869. Recovery.

Mrs. B., residing in Wallace, Steuben County, N. Y., first consulted Prof. White, July 26, 1869, in company with her son, Dr. Brasted. She stated that she was 57 years of age and the mother of 11 children, having given birth to the youngest at the age of 42,

---

* Since writing the above I learn from Prof. White that Mrs. D. died on the 27th day of February, 1870, after an attack of Peritonitis of four [4] weeks duration.

fifteen years since. She last menstruated at the age of 44. In the summer of 1868 she experienced considerable pains and tenderness in the lower portion of the bowels, which she attributed to the care necessarily bestowed upon her invalid husband. In July, 1868, she detected a small tumor in the left inguinal region. As the tumor increased in size the soreness increased in severity. She first consulted her family physician in February, 1869. From this time the tumor has given rise to a great deal of pain, and she is just recovered from an attack of peritonitis, effecting chiefly the right lumbar region. She has an ulcerated os uteri, with two polypi upon it. These are removed and local treatment administered to the ulcerated surface. She is now about the size of a woman seven months pregnant.

Diagnosis: Multilocular tumor of the left ovary.

September 26, 1869.—Prof. White received a letter bearing this date, stating that the tumor was rapidly increasing in size, and asking for its removal.

October 8.—Patient has just recovered from a second and much severer attack of peritonitis, and consults Prof. White in company with her attending physician, Dr. R. F. Parkhill. She is now much larger than a woman at full term of pregnancy, so rapid has been the growth of the tumor during the last 2½ months. That portion of the tumor occupying the left and central portion of the abdomen is hard, and yields very slightly, if at all, to pressure from without. The portion occupying the right part is softer, and presents every appearance of a large cyst filled with fluid. She is at this time seen by Drs. Gray, of Utica, and Miner, of Buffalo, who chanced to be present when she called. These gentlemen concur with Prof. White in the opinion that the case is not in every respect favorable for a successful operation, so probable is it that a large portion of the tumor is hard, and that firm adhesions attach it to the abdominal parietes and viscera. They agree, likewise, with him in the propriety of commencing ovariotomy by making an exploratory incision and if, upon examination, the adhesions can be overcome and the hard portions of the tumor can be removed, to enlarge the incision and accomplish their removal. The patient feeling that she could not long survive in her present condition, was anxious to avail herself of the only chance of life, and therefore earnestly requested the operation. Preparatory to the operation the following was prescribed: ℞ Tr. Ferri Chloride, ℥i; Sat. Sol. Potassæ Chloratis, ℥v; Sig. ʒi 4 times daily; nutritious diet was enjoined.

Operation October 25, 1869, 10 A. M.; patient cheerful; pulse 96 per minute. The following Physicians were present: Drs. R. F. Parkhill, attending Physician, and D. C. Parkhill, who administered the chloroform; Patterson of Avoca, Black of Bath, Brasted of Pultney, and Edget of Howard. The temperature of the room was raised to about 97° Fahr. Anesthesia was induced by chloroform and maintained by ether. An exploratory incision 3 inches in length was made in the linea alba, a small amount of ascitic fluid escaping. The adhesions are not so firm but that they can be readily broken away so far as the finger can reach them. A large cyst at the right of the incision, and a smaller one below, were tapped and their contents evacuated. The remaining mass was then examined and found composed of an infinite number of minute cysts, each filled with a thin, straw-colored fluid. It was, indeed, a perfect honey-comb in structure. The primary incision was then enlarged, both upward and downward; the remaining portion of the tumor so reduced in size by enucliation that it could be turned out of the abdominal cavity. It was attached to the left ovary by a thick and vascular, but not broad, pedicle. The clamp was applied; pedicle cut across and cauterized, by slowly searing it off. Upon loosening the clamp hemorrhage followed. The pedicle was, therefore, firmly enclosed in a cat-gut ligature and brought well out of the lower portion of the wound, to which it was attached by sutures in such a manner that the ligature was left on a level with the cutaneous surface. The wound was then closed by deep sutures of silver wire and superficial sutures of silk. The usual bandages of strips of adhesive plaster was then applied, the patient carried to her bed and an anodyne administered. Time occupied, 25 minutes. 3 P. M.—Pulse 92 per minute; no abdominal pain.

October 27.—Vomited three times during last night, and twice during the day; pulse 90.

October 28.—No vomiting; no tympanitis; gases escape freely from the rectum; pulse 85; tongue clean; retains nourishment well.

October 30.—No vomiting since last report; pulse 115, and soft; tongue slightly furred; kidneys acting well; urine normal.

November 1.—Pulse 100; tongue furred, but moist; discharges have loosened the adhesive straps at the lower portion of the wound; they are removed to allow free exit to the matter.

November 2.—Pulse 90, and soft; tongue clean and moist.

November 3.—Condition unchanged; wound united; superficial sutures removed. From this date forward the patient made a rapid and complete recovery.

December 7.—She is dismissed by Dr. Parkhill.

In a letter to the writer, of September 20, 1870, Dr. Parkhill states, "the case of Mrs. B. was a complete recovery. She is now in perfect health."

Case 15.—Multilocular ovarian disease; firm adhesions; tapped seven times; one or more of the cysts ruptured in a fall five years since; death in thirty hours.

Mrs. M. G. B., aet. 57, mother of four children. Last menstruated at the age of 45. The outline of the various cysts could be defined with the eye. It has a feel like a ripe water-melon.

In the winter of 1860 and 1861, Mrs. B. was afflicted with a great deal of pain in the epigastrium, which was accompanied with more or less nausea and vomiting. The following spring she first detected a small tumor in the left lumbar region. The gradual development of the tumor was accompanied with a continuation of the pain in the epigastrium, but no pain or tenderness was perceived in the location of the tumor. This condition of gradual growth of tumor, with epigastric distress, continued until November 23, 1865, when the patient experienced a severe fall, striking upon her abdomen; which, at that time, was irregularly enlarged and not quite the size of a woman at term. Immediately the whole shape of the abdomen was changed; the enlargement becoming uniform, the umbilical region becoming flattened and soft, instead of round and hard, as it had previously been. This was followed by an attack of peritonitis, from which she barely recovered. In February, 1866, she was first tapped, 8 quarts of a fluid resembling beef-brine escaping. From that time till Dec. 3, 1869, the operation had been repeated six times; the fluid at the subsequent tappings resembling that of the first in color, and only differing from it in having, at times, considerable consistency.

Following the first five operations there were no symptoms developed which were particulaıly unfavorable. Following each of the last two, however, which occurred October 8, and December 3, 1869, she became almost completely collapsed, the prostration following the last operation, particularly, being so extreme that no hopes were entertained of her recovery. This was attended at first, and its duration undoubtedly prolonged, by an excessively irritable stomach.

January 5, 1870.—Abdomen irregularly enlarged, so that the general outline of various cysts can be defined by the eye. To the feel the tumor is peculiar; having been, not inaptly, compared by Prof. White to that of a ripe water-melon. Pelvis free; patient rapidly failing in health and spirits. Her discomfort is immense. She is conscious that, unless in some manner relieved, she must soon succumb, but is extıemely hopeful of recovery by an operation.

Diagnosis: Multilocular ovarian disease involving the left ovary, and accompanied by adhesions possibly so strong that it may be impossible to detach them. Ovariotomy advised only as a dernier resort. Indeed the shock following the later operations of paracentesis was so immense as to prove well nigh fatal, and it could scarcely be expected that the prostration following ovariotomy would be less, unless the removal of the pressure caused by the tumor, and the consciousness on the part of the patient of its removal, should in some manner avert the prostration or counteract it.

Operation, January 5, 1870, the following physicians being present: Drs. Wm. H. Reynolds, S. C. Endress, Z H. Blake and F. M. Perine. Anæsthesia produced by chloroform and maintained by ether. An incision four inches long was made in the linea alba and carried through the peritoneum, which was so altered by the repeated attacks of peritonitis as to become fully one-sixth of an inch in thickness. A presenting cyst was evacuated and this exposed another, which was also tapped. The adhesions on the anterior and lateral aspects of the tumor were so extremely firm and extensive that it was necessary to enlarge the incision, both upward and downward, in order to detach them. They were, even then, detached with the utmost difficulty, well-defined thick bands of tissue uniting the tumor to the abdominal walls so that, after the tumor was turned out, the parietal layer of the peritoneum presented a very ragged aspect. Enucleation was accomplished, and, though there was no hemorrhage, it was thought safest to cauterize the extremity of the pedicle, as there were large vessels in it. Cauterization was followed by a complete arrest of the oozing. The oozing consequent upon detaching the tumor was considerable, but readily controlled by pressure and exposure. The abdominal cavity was then thoroughly sponged out, and there being absolutely no hemorrhage, the pedicle was returned and the wound closed as usual, by deep sutures of silver wire and superficial sutures of silk. The usual bandage of strips of adhesive plaster was then applied and the patient returned to her bed, with a pulse scarcely more feeble than when the operation began. Time occupied, 45 minutes; weight of tumor, 43 pounds. As anæsthesia disappeared the patient conversed as freely as her attendant would allow. During the first twelve hours she was fully as comfortable, and her symptoms were reported by the attending physician, Dr. Perine, as favorable as they had been after the last tapping. Vomiting then began and was accompanied with so great prostration that she gradually sank, and died thirty hours after the completion of the operation. There was, perhaps, something in the nature of the tumor which might have contributed to the fatal issue in this case, though no microscopic examination was made of it. Its inner walls were uneven. Dr. Perine, in a letter dated January 7, 1870, writes: "The tumor, on careful examination, presented very much the appearance of scirrhus. This opinion is concurred in by the other medical gentlemen who examined it with me."

Probably the shock attendant upon detaching the tumor contributed most largely to the fatality of the result.

Case 19.—Unilocular ovarian disease. Abdomen immensely enlarged; extensive adhesions; patient two years confined to her bed. Operation, September 1, 1870, at Newport, R. I. Recovery.

Miss F., aet. 53, has been measurably healthy for the most of her life, though never very robust. She has been troubled with enlarging abdomen for about ten years. During the first eight years the enlargement was gradual, and did not occasion very much inconvenience; but during the last two years it has been more rapid, and on account of the immense weight of the abdominal contents she has been confined to the bed and to a position on her side. She is now feeble and emaciated. Her normal weight was about 87 ℔s. The enlargement is symmetrical. Fluctuation is perfectly distinct throughout the entire abdomen in every direction. No hard portion of a tumor is anywhere perceptible. She has never been tapped. Has had considerable pain at times, but no severe peritonitis.

Diagnosis: Unilocular ovarian disease, without ascitic fluid. Operation performed September 1, 1870, 11 A. M., at Newport, R. I., by invitation of Dr. King, attending Physician. Physicians present: Drs. King, Watson and Engs, of Newport. The temperature of the room was raised to 90° Fahr. The usual anæsthetic was administered by Dr. Engs. An incision three inches in length was made in the linea alba and carried through the peritoneum, no fluid escaping. The attachments were then separated as far as the finger could reach, the cyst was tapped, and so much of its contents evacuated as to enable the operator to turn it out through the small incision made. The adhesions, though nowhere very firm, were unusually extensive—attaching the sac everywhere to the parietal layer of the peritoneum and the intestines. These were slowly and carefully detached, the sac everted and found attached by a very broad, but thin and vascular, pedicle to the right ovary. Enucleation was accomplished and, although the pulsations of an artery could be distinctly felt in the pedicle, no bleeding was present. The clamp was, however, applied and the pedicle burned across. Upon loosening the clamp hemorrhage began, and it was found necessaay to place a silver wire around the artery referred to. Oozing still continued from the abdominal walls, where the adhesions had been detached. This was arrested by pressure and exposure. The abdominal cavity was then sponged out, and, the bleeding having entirely ceased, the wound was closed in the usual manner—by sutures of silver wire and silk. The usual bandage of strips of adhesive plaster was then applied, the patient returned to her bed and given morphine—half grss. by the mouth. Time occupied, 35 minutes; weight of tumor, 84½ pounds. The tumor consisted of a single immense sac filled with a thin, aqueous fluid.

1 P. M.—Two hours later. Beyond a slight nausea from the anæsthetic, the patient is entirely comfortable. The pulse is stronger and less frequent than before the operation was commenced.

September 3.—Patient doing well; no pain.

September 5.—Continues comfortable; cheerful and sleeps well; pain readily controlled by anodynes.

September 7.—Improvement continues; rests well; takes beef tea and milk freely.

September 11.—Dressings removed; wound united; only very slight abdominal tenderness.

September 12.—Sutures removed; bowels moved by enemæ.

September 24.—Miss F. is gradually improving. She sits up an hour or more per day for the last three days, and can bear her weight on her limbs.

Dr. Rochester, in remarks concerning the diagnosis of ovarian dropsy, said that he had observed that in cases of ascites there is always resonance above the fluid, due to the intestines being crowded up under the diaphragm, while in severe cases of ovarian dropsy there was dullness over the whole of the abdomen.

Dr. White said that in some cases a spontaneous cure occurs—the contained fluid being absorbed Mrs. Cook, of Collins, some time since came to him, having ovarian dropsy well developed. Drs. Eastman and Rochester also examined the case, and it was the opinion of all that it was a true case of ovarian disease, and should be operated on. While waiting for better weather the abdomen became less extended, and finally entirely disappeared. Dr. W. also related another case which recovered without an operation, the cure following a fall on the doorstep and was undoubtedly due to a rupture of one of the sacs which contained the fluid.

Dr. Rochester said that the above cases as reported, presented many points of interest and were well worth the attentive study of the profession. The first point was the acrid character of the fluid in the first case, which was unusual; and he should not have expected as pleasant results in such a case as in one where the fluid is bland and unirritating. The next point was the existence of pregnancy in the case which occurred at Batavia, and recovery after the abortion, which certainly was not a desirable complication. And lastly, that hardness of the tumor, which is considered an unfavorable feature when an operation is to be attempted, as is shown by the excellent recovery made by another patient. Dr. R. also reported an interesting case which shows the results which sometimes follow abortion. He was called to see a woman who had formerly been a patient of his, and whom he had delivered of children. She became tired of raising a large family, and, being pregnant, had brought about an abortion, by some means, which was followed by metritis, phlegmatia dolens and pyæmia. When called to the case he was assured by the attending physician that it was one that from the severity of the symptoms, which indicated disease of the knee-joint, required amputation. Dr. R. diagnosed the disease as an abscess forming in the tissues of the thigh, which was soon confirmed by the pointing of an abscess, at the upper part of the thigh, which was opened; and it also opened spontaneously between the anus and vulva. This case adds additional proofs to the evidence already before the profession, of the extreme danger attending procured abortions.

Dr. White said that a large proportion of the diseases of women which he is called on to treat, are due to procured abortions—hypertrophy and ulceration being very common—was called on to see a case of abscess posterior to the womb, which was caused by an abortion. It is a great evil, particularly among Americans, and is steadily on the increase. Many plans have been adopted to stop it, but none that have proved effectual. The fault is in the depraved *morale* of society, which law does not and cannot reach. Although willing to do all that was in his power to abate this great crime, he was not able to suggest a remedy.

Typhoid fever, scarlatina, dysentery and bowel complaints were reported as prevailing.

Dr. White offered the following resolution, which was adopted:

*Resolved*, That all members of this Association be urgently requested to prepare and read essays on medical subjects at its meetings.

Adjourned.

Wm. C. Phelps, Secretary.

